# Mpox-Specific Neutralizing Antibodies up to 9 Months Following 1 or 2 Doses of Intradermal MVA-BN Vaccination in Sweden

**DOI:** 10.1093/ofid/ofaf657

**Published:** 2025-10-28

**Authors:** Carmen Espinosa-Gongora, Wanda Christ, Núria Mayola Danés, Claudia Eichler-Jonsson, Finn Filén, Elisabet Storgärd, Victor Westergren, Jonas Klingström, Sara Gredmark-Russ, Kari Johansen, Anna Mia Ekström, Klara Sondén

**Affiliations:** Department of Microbiology, Public Health Agency of Sweden, Solna, Sweden; ECDC Fellowship Programme, Public Health Microbiology Path, European Centre for Disease Prevention and Control, Stockholm, Sweden; Department of Microbiology, Public Health Agency of Sweden, Solna, Sweden; Department of Medicine Huddinge, Centre for Infectious Medicine, Karolinska Institutet, Stockholm, Sweden; Department of Medicine Huddinge, Centre for Infectious Medicine, Karolinska Institutet, Stockholm, Sweden; Department of Microbiology, Public Health Agency of Sweden, Solna, Sweden; Department of Infectious Diseases, Venhälsan Södersjukhuset, Stockholm, Sweden; Department of Infectious Diseases, Venhälsan Södersjukhuset, Stockholm, Sweden; Department of Infectious Diseases, Venhälsan Södersjukhuset, Stockholm, Sweden; Department of Biomedical and Clinical Sciences, Linköping University, Linköping, Sweden; Department of Medicine Huddinge, Centre for Infectious Medicine, Karolinska Institutet, Stockholm, Sweden; Department of Infectious Diseases, Karolinska University Hospital, Stockholm, Sweden; Laboratory for Molecular Infection Medicine Sweden, Umeå, Sweden; Department of Public Health Analysis and Data Management, Public Health Agency of Sweden, Solna, Sweden; Department of Global Health, Karolinska Institutet, Stockholm, Sweden; Department of Infectious Diseases, Venhälsan Södersjukhuset, Stockholm, Sweden; Department of Global Health, Karolinska Institutet, Stockholm, Sweden; Department of Microbiology, Public Health Agency of Sweden, Solna, Sweden; Department of Medicine Solna, Karolinska Institutet, Stockholm, Sweden

**Keywords:** Mpox, MVA-BN vaccine, intradermal vaccination, neutralizing antibodies, booster

## Abstract

This study assessed clade IIb monkeypox virus–specific antibody kinetics up to 9 months following intradermal vaccination for mpox, considering previous smallpox vaccination and human immunodeficiency virus status. Neutralizing antibodies waned significantly at 3 and 9 months, in line with previous reports of antibody waning after subcutaneous vaccination. Booster dosing and immunological memory need further study.

The mpox outbreak in Africa is a public health emergency. Furthermore, the population of men who have sex with men was disproportionately affected in 2022 when the disease spread in an unprecedented way in a global outbreak [1]. A recent *Lancet* report raises concerns about the increasing number of smallpox-unvaccinated individuals and the limited global vaccine supply [2]. Since 2022, Sweden has offered at-risk populations, primarily gay, bisexual, and other men who have sex with men (GBMSM), a dose-sparing intradermal-only regimen of the Modified Vaccinia Ankara–Bavarian Nordic (MVA-BN) vaccine, as 2 intradermal doses (0.1 mL) at least 28 days apart, or a single dose for individuals with prior smallpox vaccination. Sweden phased out smallpox vaccination from childhood vaccination programs around 1976. Previous evidence suggests low monkeypox virus (MPXV)–neutralizing antibody titers post–mpox vaccination [3–6] and a similar effectiveness of intradermal and subcutaneous regimens [3, 6, 7].

## METHODS

### Study Design

This was a prospective longitudinal cohort study recruiting GBMSM receiving mpox vaccination at the Venhälsan clinic at Södersjukhuset in Stockholm, Sweden. Ethical approval was obtained from the Swedish Ethical Review Authority (Reference 2022-06672) and all patients provided written informed consent.

Inclusion criteria were as follows: (1) self-reporting with mpox risk behaviors as described by the European Centre for Disease Prevention and Control [8]; (2) no known previous mpox; (3) presenting to receive the recommended MVA-BN vaccination schedule; and (4) providing their informed consent to participate in the study.

Journal data from recruited individuals provided information on birth year, human immunodeficiency virus type 1 (HIV-1, hereafter HIV) indicators, latest CD4 count results, and ongoing use of HIV preexposure prophylaxis (PrEP). The date of each vaccination dose and follow-up visits for serum sample collection was recorded as follows:

- Sample 1: mpox vaccination first dose.
- Sample 2: mpox vaccination second dose.
- Sample 3: 28 days after completing the vaccination schedule.
- Sample 4: 3 months after completing the vaccination schedule.
- Sample 5: 9 months after completing the vaccination schedule.

Additionally, samples from 14 anonymized donors (negative controls) and 19 previously mpox-infected individuals (positive controls) were included.

### Data and Sample Management

Files with personal data created at the clinic were saved on the on-premise platform, which is approved and secured for sensitive information. Only study supervisors had access. Data were pseudonymized for data analysis and stored at the Public Health Agency of Sweden. Serum samples were stored at −80°C pending analysis, labeled with pseudonyms.

### Cell Culture and Virus Propagation

Vero cells were grown in minimum essential medium (MEM) supplemented with 5% fetal bovine serum, HEPES, L-glutamine, 100 U/mL penicillin, and 100 mg/mL streptomycin.

MPXV (clade IIb) was propagated on Vero cells and titrated via the endpoint dilution assay. Cells were infected in complete MEM. After 1 hour of incubation, the virus solution was removed and fresh growth medium was added. After 4 days, the medium was replaced with fresh medium. At 5 days postinfection, when at least 50% of the cells showed visible cytopathic effect (CPE), the supernatant was collected, and the cells were harvested using a cell scraper and centrifuged at 2000*g* for 2 minutes. The cell pellets were resuspended in 500 µL medium and pipetted up and down to mix 10 times. Cell suspensions were lysed by freeze-thawing 3 times in a dry-ice ethanol bath, after which the lysates were again pipetted up and down. The lysates were then diluted in the supernatant. The pellet was discarded, and the supernatant stored at −80°C.

### Microneutralization Test

To test the neutralizing capacity of antibodies against MPXV in patient serum, heat-inactivated serum samples (30 minutes at 56°C) were diluted in 2-fold dilution series from 1:5 to 1:640 in Eagle's MEM with 5% fetal calf serum in duplicate. The dilutions were mixed with equal volumes of 2000 Tissue Culture Infectious Dose 50 (TCID_50_)/mL MPXV (100 TCID_50_ per well), resulting in a final serum dilution series from 1:10 to 1:1280. As a control, a sample containing no serum but only medium and virus was prepared. The serum–virus solutions were incubated for 1 hour at 37°C and then transferred to 96-well plates containing confluent Vero cells. After a 5-day incubation, the cells were inspected for CPE via optical microscopy. Samples were considered neutralizing if the CPE was reduced by >50% compared to the control. Results are given as the arithmetic mean of the reciprocals of the highest neutralizing dilutions of the duplicates from each sample.

### Data Analysis

Smallpox vaccination status was determined based on birth year, categorizing individuals as smallpox unvaccinated (born after 1980), smallpox vaccinated (born before 1977), or uncertain (born between 1977 and 1980). The uncertain category was created due to the inclusion of individuals originating from countries with different smallpox vaccination strategies.

Six study participants were excluded from calculations based on reference values from negative and positive controls: 4 smallpox-unvaccinated individuals suspected of previous asymptomatic infection (prevaccination titer of ≥10), and 2 smallpox-vaccinated individuals suspected of hybrid immunity (prevaccination titer ≥120).

**Figure 1.**
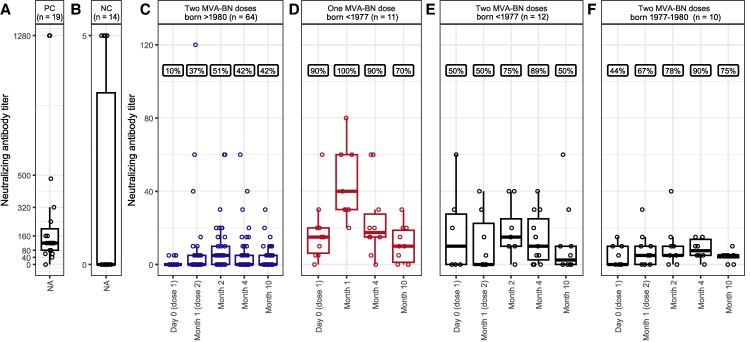
Dynamics of monkeypox virus (MPXV)–specific neutralizing antibody titer before and after intradermal administration of 0.1 mL of Modified Vaccinia Ankara–Bavarian Nordic (MVA-BN) per dose, grouped by year of birth and time point. *A* and *B*, Neutralizing antibody titer in (*A*) naturally infected individuals, used as positive controls (PC) and (*B*) anonymized blood donors born after 1980, used as negative controls (NC). NA in the x-axis of *A* and *B* indicates that no specific time point applies. *C–F*, Neutralizing antibody titer in each time point in individuals (*C*) born after 1980, receiving 2 doses of MVA-BN; (*D*) born before 1977, receiving 1 dose of MVA-BN; (*E*) born before 1977, receiving 2 doses of MVA-BN; and (*F*) born between 1977 and 1980, receiving 2 doses of MVA-BN. The percentage of individuals with detectable neutralizing antibodies is indicated inside boxes for each time point.

**Figure 2.**
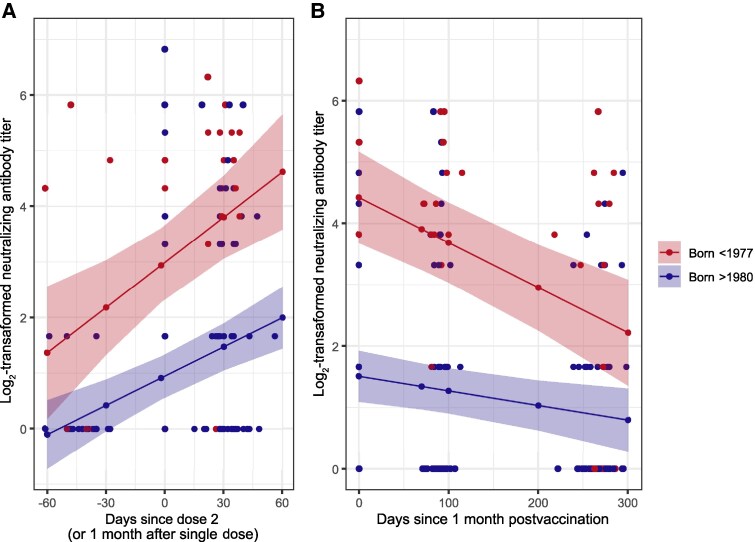
Dynamics of monkeypox virus–specific neutralizing antibody titer before and after intradermal administration of 0.1 mL of Modified Vaccinia Ankara–Bavarian Nordic (MVA-BN) per dose. *A*, Log_2_-transformed titers and linear regression model estimates across study days. Day 0 here represents the second dose for those receiving 2 doses, or the only dose for those receiving only 1 dose. Days continue counting up to 1-month follow-up of receiving the last MVA-BN dose. *B*, Days count from 1 month to 9 months after receiving the last MVA-BN dose.

Figure 1 was created with the ggplot2 package in R version 4.4.1 [9]. Mpox neutralizing antibody titers were compared between timepoints using paired Wilcoxon signed-rank tests (Figure 2). Additionally, 2 generalized linear mixed-effects models were fitted using the lme4 package in R [10] , with titer as outcome and study day as the main fixed effect. The identification of individuals was included as random effect. The first model included samples collected prior to vaccination, 1 month after the first dose, and 1 month after full vaccination (time points A, B, and C). The second model included samples from 1 month and 3 months after full vaccination (time points C, D, and E). Smallpox vaccination status, HIV status, CD4^+^ cell counts, and PrEP uptake were explored as additional fixed effects. Based on likelyhood ratio test, smallpox was the only variable included in both models in addition to study day.

## RESULTS

A cohort of 97 GBMSM was enrolled, resulting in 401 samples. Median age was 37 years (range, 23–62 years) including 64 individuals born after 1980, 23 born before 1977 (11 receiving a single MVA-BN dose), and 10 born between 1977 and 1980. Additionally, 17 were living with HIV with CD4 counts >500 cells/μL. Clinical characteristics are shown in Supplementary Table 1.

Prevaccination immunity was present in 10% of individuals born after 1980 (median titer 5), and in 90% (median titer 15) and 50% (median titer 30) of those born before 1977, receiving 1 or 2 doses, respectively (Figure 1). Immunity peaked 1 month after the last dose, with 51% of individuals born after 1980 showing detectable neutralizing antibodies (median titer 10), compared to 100% (median titer 40) and 89% (median titer 30) of those born before 1977, receiving 1 or 2 doses, respectively (Figure 2). Based on linear regression, titers increased at the first timepoint after vaccination and then declined significantly at 3 and 9 months (Figure 2). The paired Wilcoxon signed-rank test provided case-specific insights (Supplementary Table 2), with results consistent with the regression analysis, which provided greater statistical power. Despite significant waning, immunity remained higher than prevaccination levels in those born after 1980. These observations were independent of HIV status.

Negative controls, corresponding to 14 anonymized donors born after 1980, had undetectable neutralizing antibody titers (n = 10) or a titer of 5 (n = 4). Positive controls, corresponding to 19 patients with known mpox infection, had a median titer of 120; however, 1 individual displayed undetectable antibodies (Figure 1*A* and 1*B*).

## DISCUSSION

This study sheds light on several factors affecting immunogenicity to clade IIb MPXV following MVA-BN vaccination for mpox, and overall patterns where only a proportion of individuals mount a neutralizing antibody response. Antibody levels wane significantly from 4 to 9 months postvaccination. The low level of neutralizing antibody contrasts to the effectiveness, which has been reported to approximately 80% [7], but is comparable to what is seen in individuals receiving subcutaneous vaccination [3, 4, 11]. This can be seen as an indication that intradermal administration is noninferior, which would need to be confirmed in clinical trials comparing the 2 dosing strategies. The study is limited by being observational not including women, children, or immunosuppressed individuals; results should be confirmed in high-quality observational studies or clinical trials. Furthermore, the correlate of protection for mpox is unknown and the study only included mpox clade IIb.

In conclusion, individuals receiving intradermal MVA-BN vaccination developed neutralizing antibodies following a pattern similar to what has been reported in the literature for subcutaneous regimens. This information is informative for vaccine policy in the large African outbreak, where the vaccine supply is insufficient. Rapid antibody waning merits consideration of booster doses for populations at risk and underscores the need for further research on long-term protection.

## Supplementary Material

ofaf657_Supplementary_Data

## Supplementary Data

Supplementary materials are available at *Open Forum Infectious Diseases* online. Consisting of data provided by the authors to benefit the reader, the posted materials are not copyedited and are the sole responsibility of the authors, so questions or comments should be addressed to the corresponding author.
