# Supplementary material for: Mpox-Specific Neutralizing Antibodies up to 9 Months Following 1 or 2 Doses of Intradermal MVA-BN Vaccination in Sweden
Source: Open Forum Infect Dis. 2025 Oct 28;12(11):ofaf657. doi: 10.1093/ofid/ofaf657 (PMC12604009; doi:10.1093/ofid/ofaf657)
Supplement: ofaf657_Supplementary_Data [file ofaf657_supplementary_data.docx]

**Supplementary results**

**Supplementary table 1.** Demographic and clinical characteristics of the study population.

| **Characteristic** | **Overall** N = 97 | **Smallpox**  **Vaccinated** N = 23 | **Smallpox**  **Unvaccinated** N = 64 | **Uncertain smallpox vaccination** N = 10 |
| --- | --- | --- | --- | --- |
| **Birth year Median (Range)** | 1986 (1961-2000) | 1968 (1961-1976) | 1991 (1981-2000) | 1978 (1977-1980) |
| **Sex** |  |  |  |  |
| Male | 97 (100%) | 23 (100%) | 64 (100%) | 10 (100%) |
| Female | 0 (0%) | 0 (0%) | 0 (0%) | 0 (0%) |
| ***MVA-BN doses*** |  |  |  |  |
| one | 11 (100%) | 11 (100%) | 0 (0%) | 0 (0%) |
| two | 86 (100%) | 12 (14%) | 64 (74%) | 10 (12%) |
| **HIV status** |  |  |  |  |
| HIV negative | 81 (100%) | 14 (17%) | 58 (72%) | 9 (11%) |
| HIV positive | 16 (100%) | 9 (56%) | 6 (38%) | 1 (6.3%) |
| **Receiving PreP** | 45 (100%) | 9 (20%) | 32 (71%) | 4 (8.9%) |
| ***CD4 Mean (SD) among HIV positive individuals*** | 701 (137) | 739 (153) | 633 (91) | 830 (NA) |
| ***Number of samples*** |  |  |  |  |
| 2 | 5 (100%) | 0 (0%) | 4 (80%) | 1 (20%) |
| 3 | 11 (100%) | 5 (45%) | 5 (45%) | 1 (9.1%) |
| 4 | 47 (100%) | 14 (30%) | 33 (70%) | 0 (0%) |
| 5 | 34 (100%) | 4 (12%) | 22 (65%) | 8 (24%) |
| **Number of samples Mean (IQR)** | 4.13 (1) | 3.96 (0) | 4.14 (1) | 4.5 (0) |

**Supplementary table 2** . Results of comparing analysing the role of each MVA-BN vaccination dose based on paired Wilcoxon rank test, using different populations. P-values are corrected for multiple hypothesis testing (Holm).

| Question: | *Does the first dose give immunity?* | *Do two doses give immunity?* | *Does the second dose improve immunity?* | *Does immunity wane three months after the last dose in those who received two doses?* | *Does immunity wane three months after in those who received one dose?* | *Does immunity wane nine months after the last dose in those who received two doses?* | *Does immunity wane nine months after in those who received one dose?* | *Is the waned immunity at three months still higher than prior to vaccination?* | *Is the waned immunity at nine months still higher than prior to vaccination?* |
| --- | --- | --- | --- | --- | --- | --- | --- | --- | --- |
|  | A *Vs* B* | A *Vs* C* | B *Vs* C* | C *Vs* D* | B *Vs* D* (with filtering) | C *Vs* E* | B *Vs* E* | A *Vs* D* | A *Vs* E* |
| Population tested (always excluding the naturally infected) |  |  |  |  |  |  |  |  |  |
| All | Yes (p = 0.0004) | Yes (p = 0.0085) | Yes (p = 0.0118) | Yes (p = 0.0375) | Yes (p = 0.0210) | Yes (p = 0.0164) | Yes (p = 0.0170) | Yes (p = 0.0085) | No (p = 0.2842) |
| Only smallpox-unvaccinated (n=60) | Yes (p = 0.0087) | Yes (p = 0.0106) | Yes (p = 0.0139) | Yes (p = 0.0288) | (There are no smallpox-unvaccinated receiving just one dose) | Yes (p = 0.0139) | (There are no smallpox-unvaccinated receiving just one dose) | Yes (p = 0.0139) | Yes (p = 0.0139) |
| Only smallpox-vaccinated  (11 individuals received one dose, and 12 received two doses) | No (p = 0.0887) | No (p = 1.0000)** | No (p = 0.8079)** | No (p = 0.8079)** | No (p = 0.0561) | No (p = 1.0000) | Yes (p = 0.0382) | No (p = 0.8079) | No (p = 1.0000) |
| Only individuals with titre 0 or 5 prior to vaccination | Yes (p = 0.0009) | Yes (p = 0.0014) | No (p = 0.4234) | No (p = 0.7500) | No (p = 0.7500) | No (p = 0.2522) | No (p = 0.7500) | Yes (p = 0.0005) | Yes (p = 0.0027) |

* A: prior to vaccination; B: 28 days after first dose; C: 28 days after second dose (if they received a second dose); D: 3 months after the last dose (dose 2 for most, but dose 1 for those who received only one); E: 9 months after the last dose.

** Note sample size here is reduced to 12 individuals (those receiving two doses)
